# Host and Microbial Tryptophan Metabolic Profiling in Multiple Sclerosis

**DOI:** 10.3389/fimmu.2020.00157

**Published:** 2020-02-18

**Authors:** Lorenzo Gaetani, Francesca Boscaro, Giuseppe Pieraccini, Paolo Calabresi, Luigina Romani, Massimiliano Di Filippo, Teresa Zelante

**Affiliations:** ^1^Section of Neurology, Department of Medicine, University of Perugia, Perugia, Italy; ^2^Mass Spectrometry Centre (CISM), Department of Health Sciences, University of Florence, Florence, Italy; ^3^Section of Neurology, Department of Neuroscience, Agostino Gemelli Hospital, Catholic University of the Sacred Heart, Rome, Italy; ^4^Department of Experimental Medicine, University of Perugia, Perugia, Italy

**Keywords:** tryptophan, urine, signature, metabolite, multiple sclerosis, kynurenine, indole-3-propionic acid, microbiota

## Abstract

Multiple sclerosis (MS) is an autoimmune disease of the central nervous system (CNS) that is associated with demyelination and neuronal loss. Over recent years, the immunological and neuronal effects of tryptophan (Trp) metabolites have been largely investigated, leading to the hypothesis that these compounds and the related enzymes are possibly involved in the pathophysiology of MS. Specifically, the kynurenine pathway of Trp metabolism is responsible for the synthesis of intermediate products with potential immunological and neuronal effects. More recently, Trp metabolites, originating also from the host microbiome, have been identified in MS, and it has been shown that they are differently regulated in MS patients. Here, we sought to discuss whether, in MS patients, a specific urinary signature of host/microbiome Trp metabolism can be potentially identified so as to select novel biomarkers and guide toward the identification of specific metabolic pathways as drug targets in MS.

## Introduction

The levels of the essential amino acid Tryptophan (Trp) and the function of Trp derivatives have long been a subject of research interest in autoimmunity. Mammals utilize Trp for different reasons, such as protein synthesis, the release of immunomodulant catabolites, and the synthesis of the aminergic neurotransmitter serotonin, the neurohormone melatonin, several neuroactive kynuramine metabolites of melatonin, and trace amine tryptamine. Indeed, Trp is metabolized by the mammalian host cells via four different pathways, of which the most relevant is the kynurenine pathway. The other two pathways provide the transamination and decarboxylation of Trp. The hydroxylation in serotonin occurs for only 1% of dietary Trp. Interestingly, the metabolic products of the kynurenine pathway are known to have several effects on vascular system, immune system, immunotolerance, and infections.

From the time when Trp and Trp derivatives were administered in multiple sclerosis (MS) to treat autoimmunity empirically (1), many advances have been made on the knowledge of Trp metabolic functions (2). It is almost universally agreed that the catabolism of Trp has different physiological implications, such as having antimicrobial and immunomodulant properties. For all of these reasons, Trp metabolites have been largely investigated in MS (3).

From a pathophysiological point of view, MS is characterized, from its earliest stages, by the coexistence of acute focal inflammation, glial cell hyperactivation, and progressive neuro-axonal loss (4). In the relapsing-remitting phenotype of the disease, inflammatory mechanisms are prominent and are largely responsible for the clinical manifestations of the disease, which are usually transient and recurrent (4). On the contrary, the progressive phenotype of the disease, which often follows the relapsing-remitting phase, is thought to be largely sustained by neurodegenerative mechanisms, probably as the ultimate consequence of previous recurrent episodes of brain and spinal cord inflammation (5). Additionally, the development of meningeal lymphocytic infiltrates and B-cell follicle-like structures in progressive MS patients may enhance neurodegenerative phenomena (6). MS can be extremely variable between individuals, with huge differences in the frequency of episodes of focal inflammation, in the possibility of the transition from the relapsing-remitting to the progressive phenotype, in the rate of progression, and in disability outcomes (7). Among the numerous factors potentially underlying this variability, a link between environment, microbial commensals, and host immunity has been suggested (8).

The earlier findings that Trp metabolized by indoleamine-2,3-dioxygenase (IDO) along the kynurenine pathway, plays a role in the pathophysiology of neuroinflammatory and neurodegenerative disorders, led several groups of researchers to study the changes in the levels of kynurenines in plasma, urine and cerebrospinal fluid in MS patients or in mice with experimental autoimmune encephalitis (EAE), the animal model of MS (9–13).

Systemic activation of Trp metabolism may have critical effects in MS. For instance, it has been demonstrated that Trp degradation is increased in the brain during the acute phase of EAE (14). Experimental results obtained by the use of the pharmacological inhibitor of IDO (1-methyl-Trp) also support a role for this pathway in MS. Indeed, the treatment of mice with 1-methyl-Trp resulted in EAE exacerbation (14). This latter evidence might suggest a protective role of IDO metabolites in EAE, although some downstream products of the kynurenine pathway, such as quinolinic acid, may also promote neurotoxicity.

Recently, metabolomics provided new insights into the research field of MS immunopathology, showing significant promise for unraveling the sources of disease heterogeneity, for understanding the interaction between the environment and immunity, and for monitoring disease progression and response to treatment in MS patients. For instance, untargeted metabolomics has been used recently in plasma samples of EAE mice to find a signature of 44 metabolites corresponding to six major pathways that were considerably altered, including bile acid biosynthesis, taurine metabolism, tryptophan and histidine metabolism, and linoleic acid and D-arginine metabolic pathways (9). Interestingly, the signature also included various metabolites categorized under the xenobiotics class, which are normally not synthesized in the body but can be metabolized by the microbiome as equolsulphate, homostachydrine, hippurate, and a Trp-derivative, indoleacrylate, which is also excreted in urine. Besides, another Trp-derivative metabolite produced by the microbiota, indole-3-propionic acid, was found to be elevated in the plasma of EAE mice (9).

From a clinical perspective, one of the most important outcomes in MS is the risk of developing a progressive disease course (15). Indeed, while the relapsing-remitting phase can be effectively managed with immunomodulatory drugs, few treatments are available for progressive MS, and the progression of neurological disability is difficult to manage (16). In this context, Lim et al. recently wrote a paper targeted at deciphering a metabolic signature in serum to predict the transition from relapsing-remitting to progressive MS and to find a metabolic biomarker. Accordingly, they examined the role of the kynurenine pathway in MS progression and found that this pathway has a strong association with MS subtypes, correlating with disease severity scores (17).

Metabolomics has also been done on urine samples, which are readily available for analysis, and this has been used as a potential source of biomarkers in MS (18). Nuclear magnetic resonance (NMR) spectroscopy of urine allowed for the identification of metabolites that differentiated EAE-mice from healthy and MS drug-treated EAE mice (19). More recently, the metabolic profile in urine of mice bearing chronic EAE was performed with an untargeted combined metabolomics approach using gas chromatography- and liquid chromatography-mass spectrometry (GC-MS and LC-MS) (20). The authors identified eight metabolites characterizing EAE mice that are commonly found in plasma and urine and are potential biomarkers (20). Interestingly, the amino acid metabolism was primarily affected during EAE, as supported by urine analysis (20).

It is worth noting that in the diagnostic and/or therapeutic work-up of MS patients, standard urine analysis is usually performed, which makes urine sampling feasible for other investigations also. Additionally, urine is a metabolite-rich fluid that reflects the body's homeostasis and gut microbiome changes. Thus, a combined metabolomics analysis in urine, where both changes in host inflammatory/metabolic responses and in gut microbiome during MS may be highlighted, might help identifying novel biomarkers. This may provide a model to characterize pathogenic aspects of MS and to develop therapeutic approaches. We have therefore decided to perform an observational study aimed at investigating a broad panel of Trp metabolites, of both human and microbial origin, in urine samples from relapsing-remitting MS (RRMS) patients, in order to specifically investigate the possible relationship of Trp metabolites with the earliest inflammatory phase of the disease. We have compared the findings in RRMS patients to a control group of healthy individuals, and we have specifically looked for differences between MS patients and controls and for possible associations with disease characteristics.

## Materials and Methods

### Patients

Urine samples were obtained from 47 consecutive patients with RRMS and 43 healthy controls, i.e., individuals without MS or autoimmune or inflammatory diseases. Patients and healthy controls were prospectively and consecutively recruited over a 1-year period at the Section of Neurology, Department of Medicine, University of Perugia (Italy). For MS patients, inclusion criteria were: (i) a diagnosis of RRMS according to the 2010 revision of the McDonald criteria (21); (ii) no recent history of infectious disorders (i.e., <30 days before the inclusion in the study); (iii) age >18 years. The study was approved by the local Ethics Committee (# 2925/16), and patients gave informed consent for the collection of samples and subsequent analysis. The main demographic and clinical characteristics of patients were collected by experienced neurologists. For each patient, the disability level at the time of urine sampling was quantified by scoring on the Expanded Disability Status Scale (EDSS) (22). Urine samples were collected at the same time of the day (between 09:00 and 12:00) in order to avoid any potential confounding effect of diurnal rhythm. Urine samples were subsequently analyzed by laboratory technicians who were blinded to clinical data.

### Urine Analysis

Urine Trp metabolites were assessed by means of high-performance liquid chromatography-tandem mass spectrometry (HPLC-MS/MS). We used a targeted approach where a set of host or microbial metabolites derived from Trp were measured in urine. Details of HPLC-MS/MS analysis are reported in the Supplementary Methods. The following Trp metabolites and ratios were determined: (i) Trp; (ii) kynurenine; (iii) anthranilate; (iv) kynurenine/Trp (K/T) ratio; (v) kynurenine/anthranilate (K/A) ratio; (vi) 3-hydroxykynurenine; (vii) 3-hydroxyanthranilate; (viii) serotonin; (ix) tryptamine; (x) indole-3-acetic acid; (xi) indole-3-acetamide; (xii) indole-3-lactic acid; (xiii) indole-3-propionic acid.

### Statistical Analysis

Continuous variables are reported as mean ± standard deviation (SD) if normally distributed or as median, interquartile range (IQR), if non-normally distributed. Logarithmic transformation was applied to Trp metabolite values in order to reach normality, as verified with the Shapiro-Wilk test. Differences of (log) Trp metabolite values between groups were tested with the Student's *t*-test, while their association with continuous variables was tested with Pearson's correlation coefficient test. General linear models were performed for multivariable analysis. All tests were two-sided, and the significance threshold was set to *p* < 0.05. IBM SPSS Statistics software version 22 was used for statistical analysis.

## Results

### The Trp Metabolic Urinary Signature of RRMS Patients

**Figure 1:**
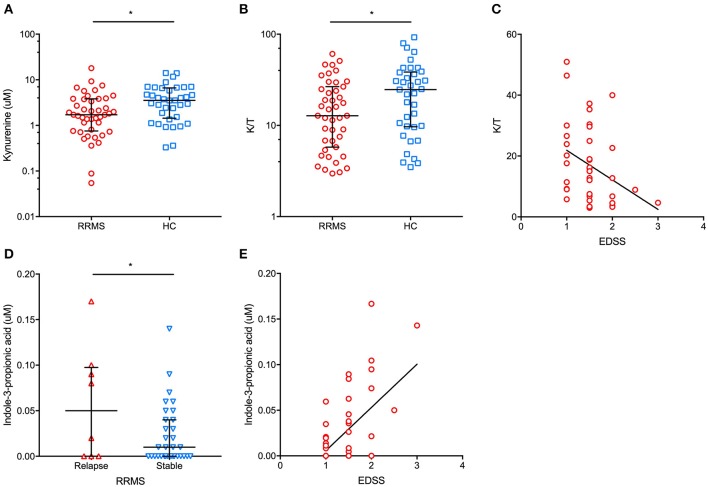
In patients with relapsing-remitting multiple sclerosis (RRMS), we found a significantly lower urinary concentration of kynurenine **(A)** (median and interquartile range are reported) and kynurenine/tryptophan (K/T) ratio **(B)** (median and interquartile range are reported) than in healthy controls (HC). Additionally, a significant negative correlation between urinary K/T and the Expanded Disability Status Scale (EDSS) score at urine sampling was found **(C)**. In RRMS patients in proximity (i.e., <30 days) to a clinical relapse, significantly higher urinary indole-3-propionic acid concentrations were found than in clinically stable RRMS patients **(D)** (median and interquartile range are reported). Moreover, urinary indole-3-propionic acid concentrations were positively correlated with the EDSS score at urine sampling **(E)**. ^*^p < 0.05.

The main demographic and clinical characteristics of RRMS patients and controls are reported in Table 1. A total of 35 patients (74.5%) were under disease modifying drugs at the time of urine sampling. In the entire cohort of MS patients and controls, females had significantly lower urinary tryptophan (*p* = 0.001), kynurenine (*p* = 0.01), anthranilate (*p* = 0.01), and serotonin (*p* = 0.01) concentrations (*p* = 0.04) than males (data not shown). After adjusting for gender, RRMS patients had a significantly lower urine concentration of kynurenine (1.4 μM, IQR: 0.5–3 μM vs. 4 μM, IQR: 1.9–6.8 μ, *p* = 0.01) and a lower K/T ratio (19, IQR: 15.5–27.5 vs. 29.8, IQR: 13.5–43, *p* = 0.04) than healthy controls (Figure 1). In contrast, no significant difference between patients and control subjects was found in the other Trp analyzed metabolites (see Materials and Methods). Within the RRMS cohort, Trp metabolites were not correlated with age and disease duration. In contrast, we found significant correlations between EDSS scores and urine concentrations of the following metabolites: (i) tryptophan (*r* = 0.5, *p* = 0.001), (ii) K/T (*r* = −0.3, *p* = 0.03), and (iii) indole-3-propionic acid (*r* = 0.5, *p* < 0.001; Figure 1). In a multivariate model taking into account age and gender, the correlations were confirmed for tryptophan (β = 0.1, *p* < 0.04), K/T (β = −0.02, *p* = 0.003), and indole-3-propionic acid (β = 4.4, *p* = 0.001). Finally, in RRMS patients, we found no difference in treated compared to untreated individuals, nor were there significant variations depending on the type of ongoing treatment.

**Table 1:** Main patient and control characteristics.

		**RRMS**	**HC**	***p*-value**
*N*^a^		47	43	ND
Age (years)^b^		31.8 ± 9.7	32.7 ± 10.6	NS
F/M		40/7	27/16	*p* = 0.02
Disease duration (years)^b^		7.5 ± 8.3	ND	ND
Ongoing therapy^a^	None Interferons Glatiramer acetate Dimethylfumarate Fingolimod Natalizumab Alemtuzumab	11 (23.4%) 15 (31.9%) 10 (21.3%) 6 (12.8%) 3 (6.4%) 1 (2.1%) 1 (2.1%)	ND	ND
EDSS^b^		1.6 ± 0.5	ND	ND
Recent relapse (<30 days)^a^		9 (19.1%)	ND	ND

a *Data are shown as number (percentage)*.

b *Data are shown as mean ± standard deviation*.

*EDSS, Expanded Disability Status Scale; HC, healthy controls; ND, not determinable; NS, not significant; RRMS, relapsing-remitting multiple sclerosis*.

In our cohort of RRMS patients, we found that urinary Trp metabolites were differently expressed in patients who had had a recent relapse (i.e., within 30 days before urine sampling). Specifically, the urine K/A ratio was significantly lower in patients with a recent relapse than in clinically stable patients (2.3 μM, IQR: 1.2–4.3 μM vs. 6.6 μM, IQR: 2.5–13.7 μM, *p* = 0.03), with a significantly higher urinary anthranilate concentration in relapsing vs. stable patients (1.1 μM, IQR: 0.5–1.8 μM vs. 0.2 μM, IQR: 0.1–0.3 μM, *p* = 0.02) (data not shown). Finally, relapsing patients had significantly higher urine indole-3-propionic acid concentrations than stable patients (0.05 μM, IQR: 0–0.1 μM vs. 0.01 μM, IQR: 0–0.04 μM, *p* = 0.04; Figure 1).

## Discussion

The pathophysiology of MS is extremely complex since it relies on the interplay between several players, such as the peripheral immune system, central nervous system resident immune cells and glial cells, and neurons (23). MS is supposed to have a multifactorial etiology, and different environmental and genetic risk factors may play a role in determining the risk of developing the disease and in driving different phenotypic disease characteristics (24). Interestingly, Trp metabolism can be influenced both by the individual genetic background and interaction with environmental factors, such as diet. A great deal of interest is now being taken in determining how microbial commensals can modulate the host immune system, since this could lead to the potential discovery of new therapeutic targets.

In this study, we found some intriguing preliminary clues that a dysbalanced human Trp metabolism may have an association with MS, a finding that is supported by the evidence that this specific metabolism plays a central role in the control of immune activation (25). Specifically, we found that in the earliest and most inflammatory phenotype of MS, i.e., RRMS, there is a specific urinary Trp metabolite signature, which is characterized by a lower concentration of kynurenine and a lower K/T ratio than in healthy controls. Additionally, K/T was negatively and independently correlated with the degree of disability at the time of urine sampling. Taken together, these findings seem to suggest that in the earliest stages of MS, a reduced Trp metabolism toward kynurenine can be found, and the lower the synthesis of kynurenine, the worse the degree of clinical impairment due to MS. Of interest, the synthesis of kynurenine via the IDO1 enzyme has been hypothesized to enhance the conversion of naïve T CD4+ cells into regulatory T cells (26), and kynurenine has shown immunoregulatory properties via the activation of the Aryl hydrocarbon Receptor (AhR) (27). Thereafter, reduced synthesis of kynurenine and a subsequent lower urinary kynurenine and K/T ratio may play a role in the pathophysiology of MS, where dysfunctional regulatory T cells favor autoimmune processes in the central nervous system (28).

From a biological point of view, once Trp is converted into kynurenine, the kynurenine pathway of Trp degradation is activated, which leads to the synthesis of a variety of compounds with both neurotoxic and neuroprotective properties that can influence MS pathology and, consequently, the degree of neurological impairment (29). Among the human urinary kynurenine downstream metabolites that we have measured in our study (i.e., 3-hydroxykynurenine and 3-hydroxyanthranilate), we did not find any significant association with the degree of disability apart from the above-mentioned negative correlation between K/T and EDSS. However, when interpreting this latter result, it should be noted that most of our patients had a low disability score. Therefore, the correlation between urinary Trp metabolites and the degree of neurological impairment deserves further confirmation on larger and more heterogeneous cohorts of MS patients, also including patients with progressive MS and with a longer disease duration.

Our findings are different from those reported for serum by Lim et al., who described higher serum K/T in MS patients than in controls (17). It is possible that this opposite result relies on the use of different biological samples (i.e., urine vs. serum), potentially reflecting different phases of Trp metabolism and/or different activities of the involved enzymes at different body sites. In this context, in order to understand the relationship between Trp metabolite concentrations in different biofluids, studies examining urine, blood, and cerebrospinal fluid in the same subjects at the same time are highly desirable.

Another possible explanation for the discrepancy between our findings and those coming from the literature might be the different characteristics of the enrolled patients. For instance, in the cohorts investigated by Lim et al., progressive MS patients were also included, and none of the patients was under disease-modifying drugs. Moreover, in the same paper, no information was provided on recent disease activity, although it can be assumed that enrolled patients had no history of recent relapses since, in the previous 3 months, they had not undergone steroid therapy (17).

The latter point could be extremely important in influencing Trp metabolite concentrations in body fluids, since we found that their urinary concentrations change in proximity to a clinical relapse, with a decrease in K/A and an increase in indole-3-propionic acid.

The decrease in K/A might either reflect a reduction in urinary kynurenine, a compound with immunoregulatory properties as discussed above, or an increase in anthranilate, or both. Of interest, anthranilate has been shown to increase in the blood in a wide range of human diseases, probably with a sort of “cleaning up” effect after an acute injury (30). Indeed, anthranilate has been associated with an antagonism to other neurotoxic kynurenines, such as quinolinic acid, as well as with a reduction in oxidative stress and in inflammatory responses (30–32). Thereafter, the decrease in urinary K/A in proximity of MS clinical relapses could reflect both a decrease in urinary kynurenine, which could enhance inflammatory responses, and an increase in urinary anthranilate, probably as a consequence of compensatory mechanisms following acute inflammation.

Also, the association between indole-3-propionic acid and MS disease activity is particularly interesting, since this molecule is one of the Trp-derived metabolites produced by the microbiota, called post-biotics (33, 34), that have been deeply investigated in recent years because of their presence in peripheral tissues and their ability to bind xenobiotic receptors. Indole-3-propionic acid is now known to contribute to changes in body weight gain on a tryptophan-rich diet (35). It may destroy indoxyl sulfate-induced expression of fibrosis and inflammation in kidney proximal tubular cells (36). More interestingly, it is also considered an antioxidant and has been reported to be neuroprotective (37). More recently, the mechanism by which the indole-3-propionic acid interacts with the mammalian host epithelial barrier has been described: the xenobiotic receptor pregnane X receptor (PXR) is, in fact, able to recognize this metabolite and reduce gut inflammation (38, 39). Interestingly, high blood levels of indole-3-propionic acid have also been found by other research groups in mice with EAE (9).

Since indole-3-propionic acid has been shown to have an antioxidant effect (40), its urinary increase may reflect an additional compensatory mechanism, driven by microbial commensals, trying to counteract the negative effects of acute inflammation. On the other hand, it is not possible to rule out a deleterious effect of indole-3-propionic acid, given the positive correlation that we have found between this Trp derivative and the degree of disability. Further studies investigating the possible immune and neuronal effects of indole-3-propionic acid are therefore needed to understand its pathophysiological role in MS.

In conclusion, although our findings are preliminary and deserve further confirmation on larger and unselected cohorts, they seem to suggest that a misbalanced human Trp metabolism is associated with MS, probably reflecting disease activity and severity. More interestingly, we found some clues that commensal microbials may interact with the host, especially in proximity to MS relapses, by synthesizing compounds such as indole-3-propionic acid.

## Future Perspectives

The study of Trp metabolites in MS is providing the scientific community with fascinating clues on the interaction between the microbiota and the human immune system in the context of autoimmune diseases. Our study, performed on urinary Trp metabolites, showed some preliminary and very interesting results, such as reduced urinary K/T in RRMS and its negative correlation with disability measures. In this sense, an altered Trp metabolism could either precede or follow autoimmune pathophysiological processes taking place in MS and could have an association with acute episodes of inflammatory activity during a chronic disease such as MS. More generally, it will be fundamental to understand if Trp metabolites are associated with a pathogenic effect and/or if they reflect the bystander activation of compensatory mechanisms to the ongoing MS-related autoimmunity. This step is required in order to proceed further in the Trp metabolism research field in search of novel potential therapeutic targets. In the near future, the study of Trp metabolism with the help of artificial intelligence and machine learning may become extremely effective in assisting medical doctors and biologists to address and solve complex diagnostic, prognostic, and therapeutic tasks. Indeed, probabilistic graphical models may be used to decipher or predict how the host and the microbiota share a common metabolic nutrient such as Trp and how this shared catabolism may be affected during immunopathology. Additionally, further studies on larger cohorts of MS patients are required in order to better investigate the correlations between Trp metabolites in different biofluids and more specific disease characteristics, such as neuroradiological and cerebrospinal fluid findings.

## Data Availability Statement

All datasets generated for this study are included in the article/Supplementary Material.

## Ethics Statement

The studies involving human participants were reviewed and approved by the Umbria Region Ethics Committee (CER), Perugia, Italy (# 2925/16). The patients/participants provided their written informed consent to participate in this study.

## Author Contributions

LG collected the samples, critically read, analyzed, and discussed the literature, and conceived the outline of the manuscript. LG, MD, and TZ wrote the manuscript. GP and FB performed mass spectrometry on urine samples. PC and LR edited the manuscript and provided valuable discussion and criticism.

### Conflict of Interest

The authors declare that the research was conducted in the absence of any commercial or financial relationships that could be construed as a potential conflict of interest.
