# Supplementary material for: Host and Microbial Tryptophan Metabolic Profiling in Multiple Sclerosis
Source: Front Immunol. 2020 Feb 18;11:157. doi: 10.3389/fimmu.2020.00157 (PMC7041364; doi:10.3389/fimmu.2020.00157)
Supplement: Supplementary file 1 [file Data_Sheet_1.docx]

**Host and Microbial Tryptophan Metabolic profiling in Multiple Sclerosis**

Lorenzo Gaetani^1^, Francesca Boscaro^2^, Giuseppe Pieraccini^2^, Paolo Calabresi^3^, Luigina Romani^4^, Massimiliano Di Filippo^1^**†**, Teresa Zelante^4^***†**

^1^ Section of Neurology, Department of Medicine, University of Perugia, Perugia, Italy

^2^ Mass Spectrometry Centre (CISM), Department of Health Sciences, University of Florence, Florence, Italy

^3^ Section of Neurology, Department of Neuroscience, Agostino Gemelli Hospital, Catholic University of the Sacred Hearth, Rome, Italy

^4^ Department of Experimental Medicine, University of Perugia, Perugia, Italy

**†These authors contributed equally to this work**

***Correspondence:** Teresa Zelante

Department of Experimental Medicine, University of Perugia, Perugia, Italy.

E-mail address: [teresa.zelante@unipg.it](mailto:teresa.zelante@unipg.it)

**HPLC-MS/MS analyses**

### **Chemicals and Reagents**

All solvents and reagents were LC-MS grade and were supplied from Sigma-Aldrich (Milano, Italia). Sodium dihydrogenphosphate-1-hydrate was from Merck (Darmstadt, Germany).

Individual stock solutions of each isotopically-labeled internal standard were prepared in methanol (MeOH) at 1 mg/mL and stored at -80°C; the internal standards were: *l*-kynurenine sulfate (ring-d4,3,3-d2, 97%+) 95% (Cambridge Isotope Laboratories, CIL, USA); *l*-tryptophan (indole-d5, 98%, CIL); indole-3-acetic acid (indole-d5, 97-98%, CIL); *dl*-3-hydroxykynurenine:HCl (^13^C_2_, 99%; ^15^N, 98%, CIL); indole-2,4,5,6,7-d5-3-acetic-2,2-d2 acid (99,2%, CDN Isotope, Point-Claire, Quebec, Canada); anthranilic-3,4,5,6-d4 acid (98%, CDN). A 200 µg/mL stock solution of each isotope-labeled internal standard was prepared in MeOH and stored at -80°C.

Individual stock solutions of unlabeled analytes were also prepared in MeOH and stored at -80°C. They were prepared at concentration of 1 mg/mL: indole-3-acetamide 98%; indole-3-carboxaldehyde 97%; indole-3-acetaldehyde-sodium bisulfite addition compound; anthranilic acid 99.5%; indol-3-acetonitrile 98%; 3-hydroxyanthranilic acid 97%; serotonin hydrochloride 98%; *l*-kynurenine; 3-hydroxy-*dl*-kynurenine. All these compounds were from Sigma-Aldrich (Milan, Italy).

Two separate working standard solutions, one containing all the isotope-labeled standards and one containing all the unlabeled standards, were prepared at 60 µg/mL in MeOH and stored at -80°C. The standard solution and the isotope-labeled standard solution were diluted at 2.5 µg/mL in water with 5 mM ammonium acetate (AmAc) and 0.2% formic acid (FoAc) (eluent A) and stored at -20°C. These solutions were used to prepare a calibration curve in the range of concentration of interest for quantitative measurements.

### **Samples Preparation**

Urine were thawed at room temperature and a 25 µl volume taken, added with 20 µl of the solution of internal standards (2.5 µg/mL) and diluted to a final volume of 100 μl with HPLC eluent A. After centrifugation, the supernatant was transferred in an autosampler vial and then injected into the HPLC-MS/MS instrument.

### **Liquid Chromatography-Tandem Mass Spectrometry**

A Dionex Ultimate 3000 HPLC system was used (Thermo Fisher Scientific) coupled to an API 3000 LC-MS/MS (AB Sciex, Toronto Canada) equipped with a Turbo Ion Spray source operating in positive ion mode. Analyst software (v.1.6.2) from AB Sciex was used for data acquisition and analysis. All MS parameters were optimized by direct infusion and source parameters (gas flows and temperatures) by flow injection. The ion source operated with ion spray voltage set at 5.5 kV, curtain gas at 8, ion source temperature at 500°C, ion source gas 7000 mL/min; collision gas was nitrogen at 3.4 _*_ 10^-5^ bar pressure. Analytes were detected using scheduled multiple reaction monitoring (MRM) acquisition; two transitions were monitored for each molecule. All the acquisition parameters are listed in **Table 1**.

| Q1  (m/z) | Q3  (m/z) | RT  (min) | analyte | DP | FP | EP | CE | CXP |
| --- | --- | --- | --- | --- | --- | --- | --- | --- |
| 225.0 | 110 | 2.6 | 3-hydroxy-kynurenine | 15 | 150 | 10 | 20 | 15 |
| 225.0 | 162 | 2.6 | 3-hydroxy-kynurenine | 15 | 150 | 10 | 20 | 15 |
| 228.2 | 210 | 2.6 | 3-hydroxy-kynurenine-^13^C_2_-^15^N | 15 | 125 | 10 | 20 | 15 |
| 228.2 | 110 | 2.6 | 3-hydroxy-kynurenine-^13^C_2_-^15^N | 15 | 125 | 10 | 20 | 15 |
| 177.0 | 160 | 5.7 | serotonine | 24 | 258 | 10 | 15 | 8 |
| 177.0 | 115 | 5.7 | serotonine | 24 | 258 | 10 | 28 | 8 |
| 209.0 | 192 | 5.5 | l-kynurenine | 23 | 203 | 10 | 17 | 11 |
| 209.0 | 94 | 5.5 | l-kynurenine | 23 | 203 | 10 | 17 | 11 |
| 215.2 | 169 | 5.3 | l-kynurenine D6 | 23 | 203 | 10 | 17 | 11 |
| 215.2 | 98 | 5.3 | l-kynurenine D6 | 23 | 203 | 10 | 17 | 11 |
| 154.0 | 136 | 6.5 | 3-hydroxy-anthranilic-acid | 15 | 140 | 10 | 15 | 8 |
| 154.0 | 80 | 6.5 | 3-hydroxy-anthranilic-acid | 15 | 140 | 10 | 35 | 4 |
| 157.1 | 139 | 6.4 | 3-hydroxy-anthranilic-acid-D3 | 15 | 140 | 10 | 15 | 8 |
| 157.1 | 83 | 6.4 | 3-hydroxy-anthranilic-acid-D3 | 15 | 140 | 10 | 35 | 4 |
| 205.1 | 146 | 7.5 | tryptophan | 30 | 200 | 10 | 30 | 15 |
| 205.1 | 188 | 7.5 | tryptophan | 30 | 200 | 10 | 30 | 15 |
| 161.0 | 144 | 8.1 | tryptamine | 36 | 250 | 10 | 29 | 7 |
| 161.0 | 117 | 8.1 | tryptamine | 36 | 250 | 10 | 31 | 7 |
| 138.1 | 120 | 9.4 | anthranilic-acid | 15 | 100 | 10 | 16 | 7 |
| 138.1 | 92 | 9.4 | anthranilic-acid | 15 | 100 | 10 | 30 | 9 |
| 142.2 | 124 | 9.3 | anthranilic-acid-D4 | 15 | 100 | 10 | 16 | 7 |
| 142.2 | 96 | 9.3 | anthranilic-acid-D4 | 15 | 100 | 10 | 30 | 9 |
| 175.1 | 130 | 9.8 | indol-3-acetamide | 19 | 143 | 10 | 16 | 9 |
| 175.1 | 158 | 9.8 | indol-3-acetamide | 19 | 143 | 10 | 25 | 9 |
| 206.1 | 160 | 10.5 | indol-3-lactic-acid | 19 | 210 | 10 | 27 | 10 |
| 206.1 | 132 | 10.5 | indol-3-lactic-acid | 19 | 210 | 10 | 30 | 10 |
| 183.1 | 136 | 10.8 | indol-3-acetic-acid D7 | 36 | 280 | 10 | 22 | 9 |
| 183.1 | 109 | 10.8 | indol-3-acetic-acid D7 | 36 | 280 | 10 | 43 | 6 |
| 176.1 | 130 | 10.9 | indol-3-acetic-acid | 36 | 280 | 10 | 22 | 9 |
| 176.1 | 103 | 10.9 | indol-3-acetic-acid | 36 | 280 | 10 | 43 | 6 |
| 146.1 | 118 | 10.8 | indole-3-carboxy-aldehyde | 25 | 250 | 10 | 25 | 7 |
| 146.1 | 91 | 10.8 | indole-3-carboxy-aldehyde | 25 | 250 | 10 | 28 | 7 |
| 190.1 | 130 | 11.2 | indol-3-propionic-acid | 30 | 143 | 10 | 18 | 8 |
| 190.1 | 172 | 11.2 | indol-3-propionic-acid | 30 | 143 | 10 | 14 | 12 |
| 157.0 | 130 | 11.9 | indol-3-acetonitrile | 15 | 180 | 10 | 18 | 9 |
| 157.0 | 117 | 11.9 | indol-3-acetonitrile | 15 | 180 | 10 | 23 | 9 |

**Table 1:** List of chromatographic retention time (RT), selected MRM parameters, declustering potential (DP), focusing potential (FP), entrance potential(EP), collision energy (CE), cell exit potential (CXP) for each measured analyte.

A Ultra AQ C18 column (100 x 2.1 mm, 3 µm; Restek, USA) was used; eluents were 5 mM AmAc in water (A) and acetonitrile (B), both containing 0.2% FoAc. The column temperature was kept at 35 °C. Chromatographic separation of the analytes was performed using a linear gradient as reported in **Table 2**; 10 µL injection volume was used. The column effluent was delivered to the mass spectrometer with no split.

| Time (min) | Flow (mL/min) | B (%) |
| --- | --- | --- |
| 0 | 0.300 | 0 |
| 11 | 0.300 | 40 |
| 13 | 0.300 | 95 |
| 21 | 0.300 | 95 |
| 22 | 0.300 | 0 |
| 33 | 0.300 | 0 |

**Table 2:** Chromatographic conditions.
